# Newspapers and Medicine

**Published:** 1900-08

**Authors:** 


A Monthly Review of Medicine and Surgery.

*EDITOR:*

WILLIAM WARREN POTTER, M. D.

All communications, whether of a literary or business nature, books for review and exchanges should be addressed to the editor: 284 FRANKLIN STREET, BUFFALO, N. Y.

VOL. XL.—LVI. AUGUST, 1900. No. 1.

### NEWSPAPERS AND MEDICINE.

A QUESTION which is beginning to seriously attract the attention of medical men throughout the country, is the alarming increase in the advertising of medicines and the practice of medicine for improper purposes in the daily newspapers. It is not so long ago that the advertising portion of the newspaper which was given up to the broad enough heading of "Medical" contained a few announcements of face bleaches, hair dyes, corn cures and the like, which if they did no good and did not effect cures or beautify the human race did not do irreparable damage in the majority of cases. Now and then there appeared an advertisement of Dr. Soandso who announced himself as a specialist in chronic and nervous diseases, but beyond that it seldom went. Now, on the contrary, one cannot pick up a daily newspaper without finding a set of medical advertisements which brazenly proclaim the trade of the abortionist and the fake specialist; hope is held out to the incurable and his money is taken when there is no hope for him and taken by the most questionable methods. Those men who set themselves up as specialists in "female troubles" do untold damage and put a premium on the crime of abortion; the newspapers which receive the advertisements of this class are morally to blame for they make it possible to point the way to these dens of robbery and quackery where mysticism reigns and terror rules supreme.

Does it need any explanation whatever to interpret the real meaning of this advertisement clipped from a Buffalo newspaper?

"Ladies who are despondent and in trouble can obtain sympathy, counsel and aid by calling on Dr.———."

The following advertisement clipped from the same newspaper, the same day has but one meaning and even the most ignorant can understand it. It means abortion and nothing else.

"Ladies.—Ladies English Monthly Regulator relieves in two to five days."

These are samples of advertisements which appear every day and are not by any means the most brazen which are carried into the homes of the people of Buffalo and every other city of the state. The newspapers of Buffalo might miss a few dollars by throwing out these filthy advertisements which appear to be on the increase, but by purging their advertising columns they would rise inestimably in the minds of a decent people who do not care to have the filth of the fakir and the bid of the abortionist served with their coffee every day.

The *Christian Herald* has shut out all medical advertisements of whatever character and has made public announcement of this fact. Referring to this the St. Paul *Medical Journal* says:

We fear that we cannot hope to see this example followed by the lay press, but we have always believed that a newspaper which announced that it would admit no filthy advertisements, no advertisements which a decent man would not be willing to read aloud to his wife and daughters, would lose nothing by so doing and would probably be the gainer by it.

Following close on this reform on the part of one of the leading denominational journals of the country a daily newspaper stepped to the front and in a terribly bitter arraignment of the "plunderers" announces that its columns are forever closed to advertisements of a sexual or venereal character. This pioneer in honestly decent journalism is the St. Paul *Daily News*, which, in referring to the matter says:

The man who trades on the ills of his fellow being, who carries on a system of highway robbery and deludes the unsuspicious and ignorant into belief in his quackery, and who worms the money from the suffering to chuckle at their credulity, is a villain by nature and has made no attempt to improve on the original plan of his conception.

And yet these scoundrels are permitted to ply their trade unhindered by the newspapers and untouched by public sentiment, because forsooth their large and showy announcements put a few dollars into the ever-reaching maw of neglectful and indifferent publications.

It is time to call a halt. It is time to rigorously enforce all legal restrictions against the existence of these plunderers; and if legal measures fail, it is the duty of the newspaper by forewarning to guard the public against those snakes in human guise. And their number is legion.

It is a moderately safe premise that an institution that promises absolute cures, without reserve, is a good place to avoid; but when an institute backs these promises by agreements, only to secure money and then to turn the cold shoulder to the poor sufferer who paid his all for treatment, it becomes nothing short of obtaining money under false pretences, and should be treated as the crime deserves.

The *Daily News* makes no sweeping charge that all advertising medical institutions are to be placed in the same category. But the *Daily News* is now unearthing the methods of the more dangerous and boldest medical fakirs, and will shortly let the full light of publicity shine upon their nefarious practises. These unscrupulous "quacks" are rascals of the first degree, and robbers in the fullest sense of the word. The *Daily News* will give the public an opportunity to discover whether this gentry can or cannot be suppressed, and whether these festering leeches whose sole thought is gain, whose every act is dictated by a wish to profit by the ills and sufferings of poor humanity, whose very ailment and physical weaknesses should entitle them to sympathy and protection, can or cannot be suppressed.

There is now an opening for a pioneer in this line in Buffalo. There are seven daily newspapers in this city and their proprietors are men of standing socially, morally and to a certain extent religiously. It will be interesting to see which of them puts the decency-in-advertising rule into effect first.

